# Peripubertal changes in circulating antimüllerian hormone levels in girls

**DOI:** 10.1016/j.fertnstert.2013.01.139

**Published:** 2013-06

**Authors:** Hany Lashen, David B. Dunger, Andy Ness, Ken K. Ong

**Affiliations:** a Reproductive and Developmental Medicine Unit, University of Sheffield, Sheffield, United Kingdom; b Department of Paediatrics and Institute of Metabolic Science, University of Cambridge, Cambridge, United Kingdom; c School of Oral and Dental Sciences, University of Bristol, Bristol, United Kingdom; d Medical Research Council Epidemiology Unit, Institute of Metabolic Science, Cambridge, United Kingdom

**Keywords:** AMH, puberty, peripubertal, ovarian, ovary

## Abstract

**Objective:**

To identify correlates and longitudinal changes in circulating antimüllerian hormone (AMH) levels as a marker of ovarian primordial follicle recruitment in normal peripubertal girls.

**Design:**

Observational study using mixed longitudinal and cross-sectional analyses.

**Setting:**

Not applicable.

**Patient(s):**

Unselected girls assessed at ages 7–11 years.

**Intervention(s):**

None.

**Main Outcome Measure(s):**

AMH, inhibin B, and FSH levels were analyzed in blood samples collected at ages 7, 9, and 11 years for longitudinal analyses and at age 8 years for cross-sectional analyses.

**Result(s):**

In the cross-sectional analysis, AMH levels at age 8 years were lower in pubertal girls (median 25.0 pmol/L, interquartile range [IQR] 16.0–33.9; n = 39) than in prepubertal girls (33.5 pmol/L, IQR 22.3–49.1; n = 342). In prepubertal girls, higher AMH levels were associated with higher inhibin B levels, lower FSH levels, and larger body mass index at age 8 years and subsequently with later age at menarche. AMH levels were unrelated to birth weight or birth length. In the longitudinal analysis, AMH levels increased between ages 7 (median 27.0 pmol/L, IQR 19.2–34) and 9 years (32.0 pmol/L, IQR 26.5–42.7), then declined between 9 and 11 years (26.5 pmol/L, IQR 19–42.25) with high intraindividual correlation in AMH levels between ages 7 and 9 years and 7 and 11 years.

**Conclusion(s):**

Measurement of circulating AMH and inhibin B levels suggests that the rate of ovarian primordial follicle recruitment increases in the prepubertal years then declines again following the onset of puberty as follicular activity pattern changes.

**Discuss:** You can discuss this article with its authors and with other ASRM members at **http://fertstertforum.com/lashenh-peripubertal-amh-puberty-ovary/**

The dimeric glycoprotein antimüllerian hormone (AMH) is produced from the granulosa cells of developing preantral and small antral ovarian follicles and may act to provide feedback inhibition of follicle development at two levels: first, by inhibiting the recruitment of further primordial follicles into the growing pool; and second, by reducing the sensitivity of antral follicles to FSH (1). High levels of AMH were reported in the follicular fluid of small antral follicles, and significantly reduced levels were reported in preovulatory follicles (2), suggesting that the production of AMH is independent from the granulosa cell number and more likely reflects the number of early antral follicles. Circulating AMH levels are considered to be a reliable marker of the rate of recruitment of primordial follicles into the growing pool, which in turn reflects the size of the ovarian reserve of primordial follicles. Accordingly, in adult women, AMH levels decline steadily with age and are highly predictive of age at menopause (3). Inhibin B also is secreted from preantral and small antral ovarian follicles, but, unlike AMH, inhibin B secretion is stimulated by FSH and its circulating levels therefore reflect both the number and the pituitary-stimulated activity of early developing follicles (4).

In children, circulating AMH levels during childhood and adolescence have recently been described in longitudinal and cross-sectional studies (5–7). These studies reported that AMH levels are not stable during childhood, but rise during infancy. The rise continues during the years leading up to puberty, roughly doubling between ages 4 and 8 years and then reaching a plateau during adolescence. The observed steady decline in AMH levels during adulthood is consistent with the dogma that a girl’s ovarian reserve of primordial follicles is established in utero and soon after birth (8) and then declines progressively until she reaches menopause (9–11). Mathematical models based on histologic studies have suggested that the highest rate of recruitment of nongrowing follicles takes place between birth and 14 years of age (11), which is synchronous with menarche in the majority of girls. However the number of subjects under the age of 14 included in that study was too small to establish what actually took place in early childhood compared with the prepubertal years. Nevertheless, the reported marked increase in AMH levels (5–7) during early childhood confirms to some extent the Wallace and Kelsey (11) findings that may indicate a process of up-regulation of such activity in the years leading up to the onset of puberty.

Although cross-sectional studies infer that AMH levels are stable between ages 8 with 25 years and then gradually decline with age (5, 6), more detailed longitudinal studies have revealed a 30% reduction in AMH levels during the first 2 years after pubertal onset (12), indicating the possible regulation of AMH production during puberty. In consideration of the potential factors that might regulate AMH production during childhood, studies have related AMH levels in children and adults to certain growth characteristics, although the findings are inconsistent. Early small studies suggested that AMH levels were higher in infants following low birthweight (BW) or high BW, compared with normal BW, suggesting antenatal altered follicular development (13). However, recent larger studies found no differences in AMH levels in prepubertal girls or young women according to birthweight or gestational age (14, 15). Furthermore, growth hormone treatment had no effect on AMH levels. In adults, lower AMH levels in obese versus normal weight has been reported in some (16, 17) but not all (18) studies. However, such relationships may be obscured by the higher AMH levels that are consistently observed in women with polycystic ovary syndrome (PCOS), as well as in their first-degree relatives, even during childhood (19, 20).

In a large sample of normal girls, we aimed to identify the hormonal and growth-related correlates of the rate of ovarian primordial follicle recruitment during peripuberty at age 8 years with the use of AMH as a marker of such activity. Furthermore, in a longitudinal study on a smaller number of subjects, we explored the temporal changes and degree of intraindividual tracking in AMH levels (as a marker of follicular recruitment activity) and inhibin B levels (as a marker of follicular development activity) in the peripubertal years.

## Materials and methods

### Population

The Avon Longitudinal Study of Parents and Children (ALSPAC) is a prospective cohort recruited from all pregnancies in three Bristol-based District Health Authorities in the United Kingdom with expected dates of delivery from April 1991 to December 1992. Out of the initial 14,541 pregnant mothers who enrolled in the ALSPAC study, there were 14,062 live births and 13,988 infants alive at 1 year. Details of antenatal data collection and measurement of body size from birth to 5 years by the ALSPAC study team is available at www.alspac.bristol.ac.uk.

Serum samples were available at age 8 years in 381 full-term (gestational age ≥37 weeks) singleton girls from a previous study in a random ALSPAC subsample (21, 22). Samples were collected in the morning after an overnight fast. In addition, the whole cohort was invited to provide venous blood samples at ages 7, 9, and 11 years, following application of a topical anesthetic cream. For the pilot longitudinal study, 32 girls who had serum samples available at all three ages (7, 9, and 11 years) were randomly (the samples from the first 32 girls in the database) chosen from the last 6 months of ALSPAC full-term births.

Fat mass at mean age 9.9 years was measured using a Lunar Prodigy Dual-energy X-ray absorptiometry scanner (GE Medical Systems Lunar). Scans were visually inspected and realigned where necessary. Analyses that included fat mass also included height and height-squared as covariables to ensure adjustment for greater relative adiposity, rather than greater fat mass as a result of greater height. Height was measured to the nearest 0.1 cm with a Harpenden stadiometer with the participant unshod.

Self-assessment of pubertal development was obtained from the girls at age 8 years. Respondents were asked to examine line drawings and accompanying descriptions of the five Tanner stages for breast size and pubic hair and to record which drawing most closely resembled their stage of development The diagrams used were previously validated (correlation with physician assessments were 0.63 for breast development, and 0.81 for pubic hair distribution) (23). Girl’s age at menarche was assessed by questionnaire at ages 11 and 13 years and categorized into three groups: <12 years (n = 65), 12–13 years (n = 74), and >13 years or “not yet” (n = 174).

Ethical approval was obtained from the ALSPAC Law and Ethics Committee and the local Research Ethics Committees. Signed consent for before-breakfast study was obtained from a parent or guardian, and verbal assent was obtained from each of the children.

### Hormone Assays

AMH was assayed in serum samples with the use of ELISA Immunotech). The sensitivity was 0.7 pmol/L (1 ng/mL = 7.14 pmol/L). The manufacturer’s intra-assay coefficient of variation (CV) based on samples assayed 12 times was ≤12.3%, and interassay CV based on samples assayed in duplicate in 11 different series was <14.2%.

FSH was assayed with the use of electrochemiluminescence immunoassay designed for use on an Elecys and Cobas immunoassay analyzer (Roche Diagnostics). The assay range was 0.1–200 IU/L, and the CV was 4.5%.

Inhibin B was assayed with the use of Gen II ELISA (Immunotech) on a Beckman Coulter analyzer. The sensitivity was 2.6 pg/mL and the CVs 4.4%–6.8%.

LH levels were below the limit of detection (0.1 IU/L) in the first 100 samples and were therefore not analyzed in further samples.

Information on the following hormone levels in the same samples collected at age 8 years were available from previous projects: IGF-1, insulin, DHEAS, androstenedione, and leptin (22, 24).

### Statistical Analysis

AMH levels at age 8 years showed a positive skewed distribution and were log-transformed to allow use of parametric tests. Multivariate linear regression was used to test the correlations between AMH levels at age 8 years and body size and hormone levels (FSH and inhibin B) at age 8 years. Adjustments were made for the child’s age at the 8 year clinic visit by entering age as a covariable. Results are displayed as betas (partial standardized correlation coefficient).

For the pilot longitudinal study, we used paired *t* tests to compare AMH levels between ages 7 and 9 years and between ages 9 and 11 years. Pearson correlation was used to analyze the correlation between AMH levels at the different ages.

## Results

### Cross-Sectional Associations at Age 8 Years

**Figure 1:**
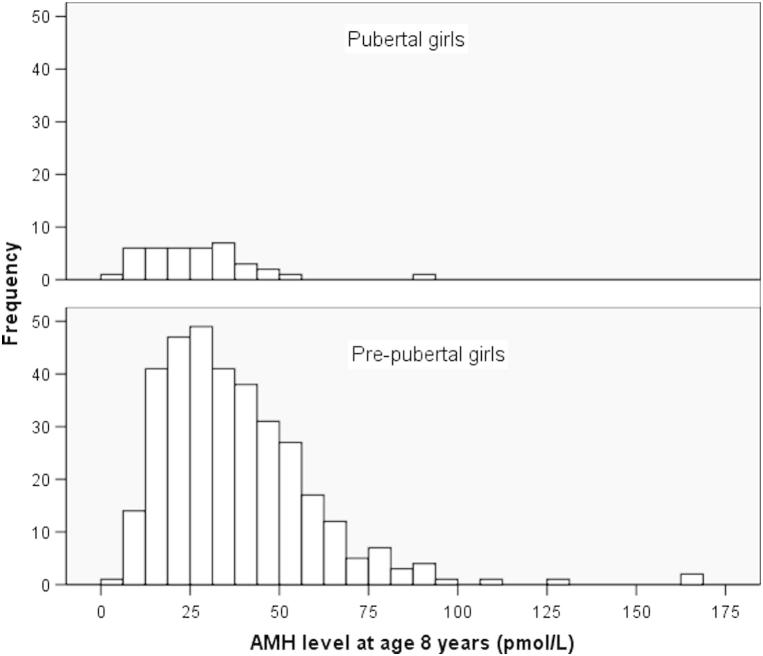
Antimüllerian hormone (AMH) levels at age 8 years in pubertal girls (median 25.0 pmol/L, interquartile range [IQR] 16.0–33.9; n = 39) and prepubertal girls (33.5 pmol/L, IQR 22.3–49.1; n = 342).

In 381 girls at age 8 years (median age 8.2, range 8.0–8.5), median AMH levels were lower in the 39 girls with self-reported pubertal breast development (25.0 pmol/L, interquartile range [IQR] 16.0–33.9) than in 342 prepubertal girls (33.5 pmol/L, IQR 22.3–49.1; *P*<.0001; Fig. 1).

Within prepubertal girls, higher AMH levels were also associated with: higher inhibin B levels, lower FSH levels, and lower BMI at age 8 years; with lower fat mass index at 9 years (Table 1); and with subsequent later age at menarche (*P*=.03). AMH levels at age 8 years were unrelated to birth weight or gestational age (*P*>.4; data not shown).

Five (1.3%) of 381 girls (three prepubertal) had very low AMH levels of <8 pmol/L, which is the cutoff used to identify ovarian failure in girls with Turner syndrome (25). These five girls also had lower levels of inhibin B (median 5.9 pmol/L, range 2.8–7.4) compared with other girls (median 14.7 pmol/L, IQR 9.4–23.9), but no differences in FSH levels (data not shown).

### Pilot Longitudinal Study

**Figure 2:**
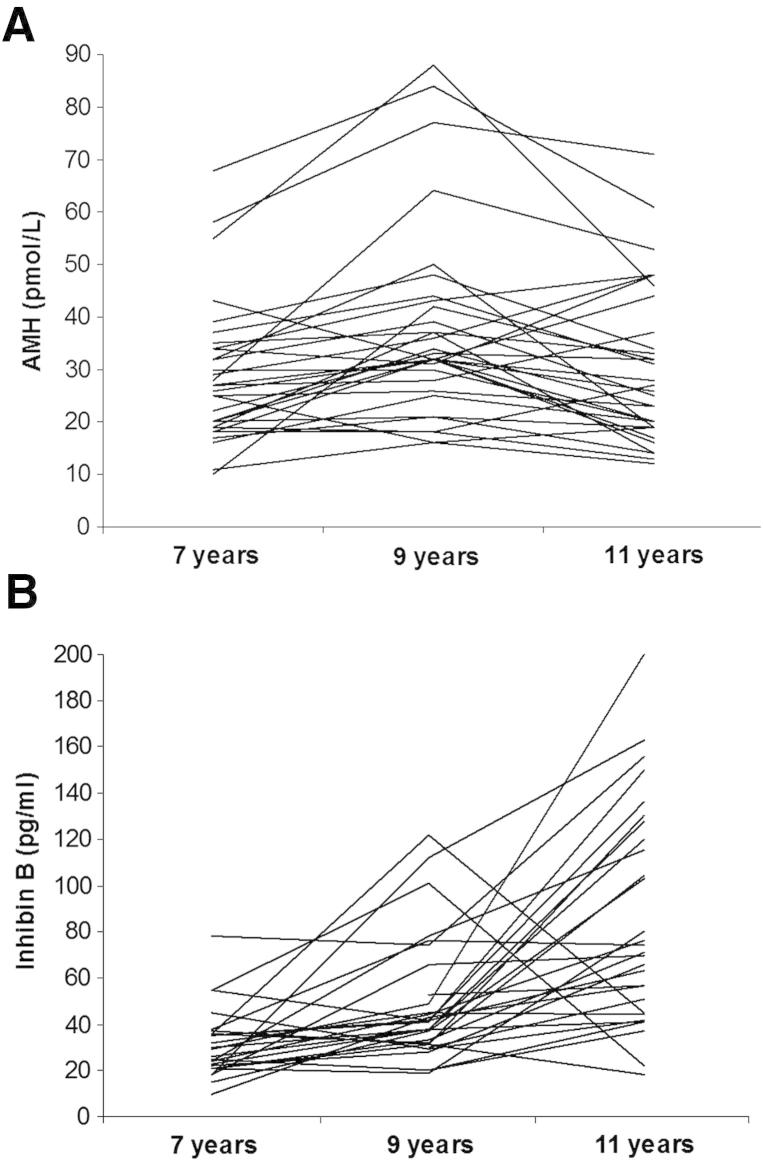
Longitudinal changes in circulating (**A**) antimüllerian hormone (AMH) levels and (**B**) inhibin B levels, between ages 7, 9, and 11 years old in 32 girls. Each line represents an individual girl.

In 32 girls with longitudinal data, AMH levels rose between ages 7 years (median 27.0 pmol/L, IQR 19.2–34) and 9 years (32.0, IQR 26.5–42.7; paired *t* test: *P*<.0001), and then declined by a similar amount up to age 11 years (26.5, IQR 19–42.25; *P*=.003; Fig. 2). Despite these changes, there was strong intraindividual tracking in AMH levels, both between 7 and 9 years (Pearson coefficient: *r* = 0.81; *P*<.001) and between 9 and 11 years (*r* = 0.73; *P*<.001). In contrast, inhibin B levels increased continuously from ages 7 years (median 27.5 pg/mL, IQR 21.0–36.2) to 9 years (41.0, IQR 31.0–59.5; paired *t* test: *P*=.002) and 11 years (72.5, IQR 46.5–126.0; paired *t* test: *P*=.0007), but showed little intraindividual tracking between 7 and 9 years (*r* = 0.26; *P*=.2) or between 9 and 11 years (*r* = 0.13; *P*=.5).

Baseline inhibin B levels at age 7 years were positively associated with early changes in AMH between 7 and 9 years (correlation coefficient = 0.45; *P*=.01) and inversely associated with later changes in AMH between 9 and 11 years (*r* = 0.52; *P*=.003).

## Discussion

We measured circulating AMH levels as a marker of the rate of recruitment of primordial follicles. Our findings of complex changes in, and cross-sectional associations with, AMH levels indicate intriguing changes in the rate of ovarian follicle recruitment during the peripubertal years, which correspond to the timing of menarche.

### Prepubertal Rise in AMH Levels

Our observation of a longitudinal 20% rise in AMH levels during the 2 years between ages 7 and 9 years is consistent with the inferred doubling in AMH levels between ages 4 and 8 years in cross-sectional data (5–7). Together, these findings suggest that the rate of recruitment of “quiescent” primordial follicles into “active” preantral follicles that produce AMH is not a constant, nor simply a function of primordial follicle numbers, but rather increases steadily as girls approach the onset of puberty. This conclusion is consistent with a recent review and analysis of cross-sectional data on primordial follicle numbers from autopsy samples, which estimated that the rate of follicle recruitment increases with age during childhood, despite declining total primordial follicle numbers (7).

The longstanding concept that the pituitary-gonadal axis is inactive from late infancy until the abrupt reawakening of pituitary sensitivity to GnRH has been questioned in recent years, with studies reporting gradual rises in the levels of E_2_ and inhibin B during the prepubertal years (25). It is not possible to say whether a gradual increase in ovarian follicle activity facilitates or even triggers the onset of puberty, but the concept is plausible.

### Reduction in AMH Levels with Puberty

Our cross-sectional finding that AMH levels were ∼25% lower in pubertal compared to prepubertal girls is consistent with our pilot longitudinal data showing a 20% reduction in AMH levels between ages 9 and 11 years, and confirms other recent longitudinal data (12). The onset of puberty is characterized in the ovary by the progression of small antral follicles to antral follicles, which do not produce AMH (1), and this could contribute to the reduction in circulating AMH levels. Furthermore, Kelsey et al. recently estimated that the rate of primordial follicle recruitment peaks during adolescence and then declines (7); our findings refine the timing of such decline in follicle recruitment to the onset of puberty. In contrast to AMH, inhibin B levels increased by 49% between ages 7 and 9 years and increased by a further 76% between ages 9 and 11 years. We consider that the divergence between AMH and inhibin B levels beyond age 9 years reflects the falling rate of primordial follicle recruitment as FSH-stimulated follicle activity increases with maturation of the pituitary-gonadal axis.

Associations between AMH levels at 8 years and age at menarche, and between baseline inhibin B levels and later changes in AMH, suggest that prepubertal ovarian follicle activity may predict the timing of pubertal onset and completion. However, this needs to be confirmed in larger longitudinal studies, ideally with the use of objective assessments of pubertal timing. Longer follow-up is needed also to show whether the close intraindividual tracking in AMH levels during adolescence continues into adult life.

### Other Associations

We detected other associations—between lower circulating AMH levels and higher BMI, higher leptin levels, and earlier timing of menarche—that are less easy to interpret in the present cross-sectional sample. Such observations appear to contrast with the reported higher AMH levels in women with PCOS and their first-degree relatives in childhood (19, 20). PCOS is a condition closely related to excess weight gain and hyperandrogenism. In the ALSPAC study we have previously shown that DHEAS and androstenedione levels are associated with low birthweight and accelerated postnatal weight gain (22). However, we could not detect any relationship between AMH levels and levels of these androgens, nor with birthweight, in the 8-year-old girls. The paradoxic findings of elevated AMH levels in obese PCOS women yet reduced AMH levels in prepubertal girls with higher BMI may reflect genetic/environmental disturbances in the regulation of antral follicular development in PCOS.

Indeed, the influence of sex steroids on circulating AMH levels remains debated. Initial studies suggested that AMH levels were stable, as they showed little intracycle variation and were not affected by administration of steroidal or gonadatropic hormones (26, 27). However, recent studies have reported that younger women show greater intraindividual fluctuations in AMH levels during the menstrual cycle (28). Understanding the determinants of AMH levels requires further experimental approaches as well as analyses of large longitudinal cohort studies during childhood and adolescence with measures of pubertal timing and sex hormone levels.

In conclusion, complex changes in serum AMH and inhibin B levels in girls during the peripubertal years may reflect changes in the rate of ovarian follicle recruitment and follicle activity, respectively. The rise in AMH and inhibin B levels during prepubertal childhood supports the intriguing concept that increasing ovarian primordial follicle recruitment and follicle activity precedes the onset of central puberty. The subsequent divergence in AMH and inhibin B levels following the onset of puberty may reflect the falling rate of primordial follicle recruitment when FSH-stimulated follicle activity increases. Despite these marked longitudinal changes, AMH levels tracked strongly within individuals during the peripubertal years.

## Figures and Tables

**Table 1:** Median values and cross-sectional associations with antimüllerian hormone (AMH) levels at age 8 years in prepubertal girls.

	Median, IQR	Correlation		n
		Beta	*P* value	
Weight (kg)	27, 25–31	−0.10	.07	341
Height (cm)	130, 126–133.5	−0.02	.8	341
BMI (kg/m^2^)	16.3, 15.2–17.6	−0.12∗	.03	341
Fat mass index (at age 9 y)	4.3, 3.2–5.8	−0.12∗	.04	316
Leptin (ng/mL)^a^	4.9, 3.3–8.0	−0.12∗	.02	341
Insulin (mU/L)^a^	5.2, 3.4–8.1	−0.03	.6	341
DHEAS (mcg/dL)^a^	16.9, 10.1–33.7	−0.02	.7	298
Androstenedione (ng/dL)^a^	53.4, 36.9–71.1	0.03	.4	297
IGF-I (ng/mL)	150, 117.3–181	−0.09	.3	164
FSH (IU/L)	1.2, 0.8–1.7	−0.32∗∗	<.0001	339
Inhibin B (pg/mL)	15.1, 9.3–24.9	0.41∗∗	<.0001	339

*Note:* Beta: standardized partial regression coefficient, adjusted for age at AMH measurement.

∗ *P*<.05.

∗∗ *P*<.005.

a These variables showed positively skewed distributions; therefore, correlations were analyzed on log-transformed values.

## References

[1] Broekmans F.J., Visser J.A., Laven J.S., Broer S.L., Themmen A.P., Fauser B.C. (2008). Anti-mullerian hormone and ovarian dysfunction. Trends Endocrinol Metab.

[2] Andersen C.Y., Byskov A.G. (2006). Estradiol and regulation of anti-mullerian hormone, inhibin-A, and inhibin-B secretion: analysis of small antral and preovulatory human follicles’ fluid. J Clin Endocrinol Metab.

[3] Broer S.L., Eijkemans M.J., Scheffer G.J., van Rooij I.A., de Vet A., Themmen A.P. (2011). Anti-mullerian hormone predicts menopause: a long-term follow-up study in normoovulatory women. J Clin Endocrinol Metab.

[4] Robertson D.M. (2012). Inhibins and activins in blood: predictors of female reproductive health?. Mol Cell Endocrinol.

[5] Hagen C.P., Aksglaede L., Sorensen K., Main K.M., Boas M., Cleemann L. (2010). Serum levels of anti-mullerian hormone as a marker of ovarian function in 926 healthy females from birth to adulthood and in 172 Turner syndrome patients. J Clin Endocrinol Metab.

[6] Lie Fong S., Visser J.A., Welt C.K., de Rijke Y.B., Eijkemans M.J., Broekmans F.J. (2012). Serum Anti-mullerian hormone levels in healthy females: a nomogram ranging from infancy to adulthood. J Clin Endocrinol Metab.

[7] Kelsey T.W., Anderson R.A., Wright P., Nelson S.M., Wallace W.H. (2012). Data-driven assessment of the human ovarian reserve. Mol Hum Reprod.

[8] Forabosco A., Sforza C. (2007). Establishment of ovarian reserve: a quantitative morphometric study of the developing human ovary. Fertil Steril.

[9] Block E. (1952). Quantitative morphological investigations of the follicular system in women; variations at different ages. Acta Anat (Basel).

[10] Sforza C., Ranzi A., Ferrario V.F., Forabosco A. (2004). Growth patterns of human ovarian volume during intrauterine and postnatal organogenesis. Early Hum Dev.

[11] Wallace W.H., Kelsey T.W. (2010). Human ovarian reserve from conception to the menopause. PLoS One.

[12] Hagen C.P., Aksglaede L., Sorensen K., Mouritsen A., Andersson A.M., Petersen J.H. (2012). Individual serum levels of anti-mullerian hormone in healthy girls persist through childhood and adolescence: a longitudinal cohort study. Hum Reprod.

[13] Sir-Petermann T., Marquez L., Carcamo M., Hitschfeld C., Codner E., Maliqueo M. (2010). Effects of birth weight on antimullerian hormone serum concentrations in infant girls. J Clin Endocrinol Metab.

[14] Lem A.J., Boonstra V.H., Renes J.S., Breukhoven P.E., de Jong F.H., Laven J.S. (2011). Anti-mullerian hormone in short girls born small for gestational age and the effect of growth hormone treatment. Hum Reprod.

[15] Kerkhof G.F., Leunissen R.W., Willemsen R.H., de Jong F.H., Visser J.A., Laven J.S. (2010). Influence of preterm birth and small birth size on serum anti-mullerian hormone levels in young adult women. Eur J Endocrinol.

[16] Steiner A.Z., Stanczyk F.Z., Patel S., Edelman A. (2010). Antimullerian hormone and obesity: insights in oral contraceptive users. Contraception.

[17] Piouka A., Farmakiotis D., Katsikis I., Macut D., Gerou S., Panidis D. (2009). Anti-mullerian hormone levels reflect severity of PCOS but are negatively influenced by obesity: relationship with increased luteinizing hormone levels. Am J Physiol Endocrinol Metab.

[18] Sahmay S., Usta T., Erel C.T., Imamoglu M., Kucuk M., Atakul N. (2012). Is there any correlation between amh and obesity in premenopausal women?. Arch Gynecol Obstet.

[19] Villarroel C., Merino P.M., Lopez P., Eyzaguirre F.C., Van Velzen A., Iniguez G. (2011). Polycystic ovarian morphology in adolescents with regular menstrual cycles is associated with elevated anti-mullerian hormone. Hum Reprod.

[20] Sir-Petermann T., Codner E., Maliqueo M., Echiburu B., Hitschfeld C., Crisosto N. (2006). Increased anti-mullerian hormone serum concentrations in prepubertal daughters of women with polycystic ovary syndrome. J Clin Endocrinol Metab.

[21] Ong K.K., Petry C.J., Emmett P.M., Sandhu M.S., Kiess W., Hales C.N. (2004). Insulin sensitivity and secretion in normal children related to size at birth, postnatal growth, and plasma insulin-like growth factor-I levels. Diabetologia.

[22] Ong K.K., Potau N., Petry C.J., Jones R., Ness A.R., Honour J.W. (2004). Opposing influences of prenatal and postnatal weight gain on adrenarche in normal boys and girls. J Clin Endocrinol Metab.

[23] Rubin C., Maisonet M., Kieszak S., Monteilh C., Holmes A., Flanders D. (2009). Timing of maturation and predictors of menarche in girls enrolled in a contemporary British cohort. Paediatr Perinat Epidemiol.

[24] Thankamony A., Ong K.K., Ahmed M.L., Ness A.R., Holly J.M., Dunger D.B. (2012). Higher levels of IGF-I and adrenal androgens at age 8 years are associated with earlier age at menarche in girls. J Clin Endocrinol Metab.

[25] Crofton P.M., Evans A.E., Groome N.P., Taylor M.R., Holland C.V., Kelnar C.J. (2002). Dimeric inhibins in girls from birth to adulthood: relationship with age, pubertal stage, FSH and oestradiol. Clin Endocrinol (Oxf).

[26] Mohamed K.A., Davies W.A., Lashen H. (2006). Antimullerian hormone and pituitary gland activity after prolonged down-regulation with goserelin acetate. Fertil Steri.

[27] van den Berg M.H., van Dulmen–den Broeder E., Overbeek A., Twisk J.W., Schats R., van Leeuwen F.E. (2010). Comparison of ovarian function markers in users of hormonal contraceptives during the hormone-free interval and subsequent natural early follicular phases. Hum Reprod.

[28] Overbeek A., Broekmans F.J., Hehenkamp W.J., Wijdeveld M.E., van Disseldorp J., van Dulmen–den Broeder E. (2012). Intra-cycle fluctuations of anti-Mullerian hormone in normal women with a regular cycle: a re-analysis. Reprod Biomed Online.

